# Cervical Cancer Screening Service Uptake and Associated Factors among Age Eligible Women in Mekelle Zone, Northern Ethiopia, 2015: A Community Based Study Using Health Belief Model

**DOI:** 10.1371/journal.pone.0149908

**Published:** 2016-03-10

**Authors:** Hinsermu Bayu, Yibrah Berhe, Amlaku Mulat, Amare Alemu

**Affiliations:** 1 Department of Midwifery, College of Health Sciences, Mekelle University, Mekelle, Ethiopia; 2 Department of Obstetrics and gynaecology, College of Health Sciences, Mekelle University, Mekelle, Ethiopia; 3 Department of Midwifery, College of Health Sciences, Bahirdar University, Bahirdar, Ethiopia; Rudjer Boskovic Institute, CROATIA

## Abstract

**Introduction:**

Cervical cancer is the third most common cancer among women worldwide, with about 500,000 new patients diagnosed and over 250,000 deaths every year. Cervical cancer screening offers protective benefits and is associated with a reduction in the incidence of invasive cervical cancer and cervical cancer mortality. But there is very low participation rate in screening for cervical cancer among low and middle-income countries.

**Objective:**

This study aimed to determine cervical cancer screening service uptake and its associated factor among age eligible women in Mekelle zone, northern Ethiopia, 2015.

**Methods:**

A community based cross-sectional study was conducted in Mekelle zone among age eligible women from February to June 2015. Systematic sampling technique was used to select 1286 women in to the study. A pre-tested structured questionnaire was used to collect relevant data. Data was entered and cleaned using EPINFO and analyzed using SPSS version 20 software package. Bivariate and Multivariate logistic regression was performed to assess association between dependent and independent variables with 95% CI and p-value less than 0.05 was set for association.

**Results:**

The study revealed that among 1186 age eligible women, only 235(19.8%) have been screened for cervical cancer. Age (AOR = 1.799, 95%CI = 1.182–2.739), history of multiple sexual partners (AOR = 1.635, 95%CI = 1.094–2.443), history of sexually transmitted disease (AOR = 1.635,95%CI = 1.094–2.443), HIV sero status (AOR = 5.614, 95%CI = 2.595–12.144), perceived susceptibility to cervical cancer (AOR = 2.225, 95%CI = 1.308–3.783), perceived barriers to premalignant cervical lesions screening (AOR = 2.256, 95%CI = 1.447–3.517) and knowledge on cervical cancer and screening (AOR = 2.355, 95%CI = 1.155–4.802) were significant predictors of cervical cancer screening service uptake.

**Conclusion:**

Magnitude of cervical cancer screening service uptake among age eligible women is still unacceptably low. Age of the women, history of multiple sexual partners and sexually transmitted disease, HIV sero-positivity, Knowledge, Perceived susceptibility and Perceived Barrier were important predictors of cervical cancer screening service uptake.

## Introduction

Cervical cancer is the most catastrophic malignancy of the cervix, the lower opening of uterus and it is the third most common cancer (after breast cancer and colorectal cancer) among women worldwide. About 500,000 new patients are diagnosed with cervical cancer annually of which over 250,000 die [1].

Sub-Saharan Africa contributed more than 85% of global burden of cervical cancer. It is a major cause of morbidity and mortality among women in resource-poor settings, especially in Africa [1, 2].

Of nearly 22 million Ethiopian women over the age of 15, approximately 7,600 are diagnosed with cervical cancer and roughly 6,000 women die of the disease each year. On the other hand, the incidence and mortality from cervical cancer in Ethiopia is 26.4 and 18.4/100,000 respectively. These figures are probably lower than the actual number of cases, given the low level of awareness, cost, and limited access to screening services and lack of a national cancer registry [2, 3].

Cervical cancer screening offers protective benefits and is associated with a reduction in the incidence of invasive cervical cancer and cervical cancer mortality [4]. The World Health Organization (WHO), United States Preventive Services Task Force (USPSTF) and the American Cancer Society (ACS) recommended that all age eligible women should have cervical cancer screening at least once every three years [5, 6, 7].

Ethiopia adopted the WHO recommendation and recommended women to begin cervical cancer screening at age of thirty five and above or three years past coitarchea at least once every three years. The “see and treat” strategy is being applied using Visual Inspection under Acetic acid (VAI) as screening method and cryotherapy as a treatment option [8].

But women in low and middle-income countries have a low participation rate in screening for cervical cancer. For example, only 6%, 12%, and 8.3% of age eligible women in South Africa, Bhutan, and Nigeria have participated in cervical cancer screening service uptake respectively [9, 10, 11].

Surprisingly in Ethiopia, only 1% of age eligible women receive effective screening for cervical cancer and 90% of women have never had a pelvic examination at all [12].

Therefore, the main purpose of this research was to identify factors affecting cervical screening service uptake and recommend ways to increase screening uptake by the community.

## Methods

A community based cross-sectional study was conducted in Mekelle zone from February to June 2015 among age eligible women. The zone is comprised of two towns, Kuwiha and Mekelle, the capital city of Tigray regional state. The zone has two cervical cancer screening centers, one at Mekelle Hospital and another in Ayder Referral Hospital. The study population includes all age eligible women (age ≥ 21 years) who have been living in Mekelle zone at least for six months [5].

The sample size was determined using a single population proportion formula considering the following assumptions: 8.3% proportion of women who underwent cervical cancer screening [11] and 5% level of significance (α = 0.05). The final sample size was adjusted for a non- response rate of 10% and the total sample arrived at was 1286.

Systematic sampling technique was used to select participants of the study. The total sample size calculated for the study (1286) was distributed to the kebeles in the zone using proportional allocation based on the estimated number of households.

Data was collected by six diploma midwives through face-to-face interviews using a structured and pre-tested questionnaire after one day training was given to them together with their BSc midwife supervisor.

Data was entered using EPIFO and analysis was performed using SPSS version 20.0. Variables reaching a p-value of 0.2 on bivariate analysis were included in multiple logistic regression analysis and p-values of less than 0.05 were taken to represent significance. The degree of association between the independent and dependent variables was analysed using odds ratios with 95% confidence intervals.

Ethical clearance was obtained from the Institutional Review Board (IRB) of Mekelle University, College of Health Sciences. A formal letter of cooperation was sent to Mekelle Zone Health Bureau and a formal letter of permission was obtained. Finally, a written informed consent was obtained from each age eligible woman.

## Results

### Socio-demographic characteristics

Among the 1286 sampled age eligible women, 1186 responded to the questionnaire, giving a response rate of 92%. The mean age of the study participants was 31.3 years (31.1±9.3SD). The majority of women were Tigrian 969(81.7%), married 768(64.8%) and Orthodox Christian 756(63.7%). One thousand forty one (87.7%) of the women have attended at least primary education (Table 1).

**Table 1:** Socio-demographic Characteristics of age eligible women Mekelle Zone (n = 1186) Mekelle zone, June, 2015.

Characteristics	Frequency (%)
**Age of mothers during the interview (mean, SD = 31.3 years, ±9.3SD)**	
21–29	614(51.8)
30–39	415(35.0)
40–49	81(6.8)
≥50	76(8.4)
**Marital status**	
Married	768(64.8)
Single	173(14.6)
Divorced	178(15.0)
Widowed	67(5.6)
**Religion**	
Orthodox	756(63.7)
Muslim	423(35.7)
Protestant	4(.3)
Others*	3(.3)
**Educational status**	
No formal education	145(12.2)
Primary education	386(32.5)
Secondary &high school	362(30.5)
Collage and above	293(24.7)
**Ethnicity**	
Tigre	969(81.7)
Amhara	217(18.3)
**Occupation**	
House wife	550(46.4)
Self-employee	379(32.0)
Gev-employee	181(15.3)
Others**	76(6.4)
**House hold income**	
<900	305(25.7)
900–1600	302(25.5)
1600–2700	291(24.5)
>2700	288(24.3)

* Joba, Catholic and 7^th^ day Adventist.

** student, daily laborer and shop kipper

### Reproductive characteristics

Two hundred seven women (17.5%) had sexual intercourse for the first time at the age of sixteen or below. Majority of the women, 978 (82.5%) have given birth at least once while 432(36.4%) had a history of combined oral contraceptive use.

Three hundred eighty one (32.1%) participants admitted that they had history of multiple sexual partners during the last three years. One thousand one hundred eighteen (94.3%) of participants had HIV test and 65(5.5%) of them were found to be positive. Fifteen (1.3%) and 116(9.8%) participants have history of smoking and STD respectively (Table 2).

**Table 2:** Reproductive Characteristics of age eligible women in Mekelle Town, northern Ethiopia, June, 2015.

FACTORS	FREQUENCY (%)
**Age at first sex**	
≤16	207(17.5)
>16	979(82.5)
**Multiple sexual partner**	
No	805(67.9)
Yes	381(32.1)
**Ever had history of smoking**	
No	15(1.3)
Yes	1171(98.7)
**Ever use of COC pills**	
No	754(63.6)
Yes	432(36.4)
**Ever had history of STD**	
No	1070(90.2)
Yes	116(9.8)
**HIV test**	
No	68(5.7)
Yes	1118(94.3)
**Sero status**	
Negative	1052(88.7)
Positive	65(5.5)
**Ever had gave birth**	
No	208(17.5)
Yes	978(82.5)

### Knowledge, perception and practice of cervical cancer screening

Majority of the respondents (85.8%) have at least heard of cervical cancer and its screening. One thousand one hundred nine (93.5%) participants knew that cervical cancer is a killer if not detected early, but only 292(24.6%) knew that they can develop a premalignant condition of the cervix without any symptoms.

With regard to their perceptions, 1145(96.5%) of the participants agreed that cervical cancer can be severe and may be hazardous to their health. Therefore, majority of the participants 1151(97.0%) agreed precancerous cervical screening is beneficial for their wellbeing. However, only 822 (69.3%) participants felt that they themselves are susceptible to develop the condition. Furthermore, only 235 (19.8%) underwent screening during the last three years (Table 3).

**Table 3:** Distribution of Knowledge and Perception of cervical cancer screening among Age Eligible Women Mekelle Zone, Ethiopia, June, 2015.

Variables	Frequency (%)
**Ever heard about cervical cancer**	
No	168(14.2)
Yes	1018(85.8)
**Ever heard about cervical cancer screening**	
No	168(14.2)
Yes	1018(85.8)
**Precancerous cervical screening in Mekelle**	
No	143(12.1)
Yes	1043(87.9)
**Premalignant cervical dysplasia can happen without symptoms**	
No	894(75.4)
Yes	292(24.6)
**Cervical cancer is a killer if not detected early**	
No	77(6.5)
Yes	1109(93.5)
**Any reproductive age woman is susceptible to develop cervical cancer.**	
I agree	865(72.9)
Indifferent	187(15.8)
I disagree	134(11.3)
**Like any women, I am susceptible to develop cervical cancer.**	
I agree	822(69.3)
Indifferent	175(14.8)
I disagree	189(15.9)
**Cervical cancer can be severe and may be hazardous**	
I agree	1145(96.5)
Indifferent	22(1.9)
I disagree	19(1.6)
**Precancerous cervical screening to may be beneficial my wellbeing**	
I agree	1151(97.0)
Indifferent	24(2.0)
I disagree	11(.9)
**Over all knowledge**	
Not Knowledgeable	207(17.5)
Knowledgeable	979(82.5)
Even, if I see screening is beneficial, I can’t do so	
I agree	734(61.88)
Indifferent	344(29.0)
I disagree	108(9.1)
**Screened at least once during the last three years**	
Yes	235(19.8)
No	951(80.2)

**Fig 1:**
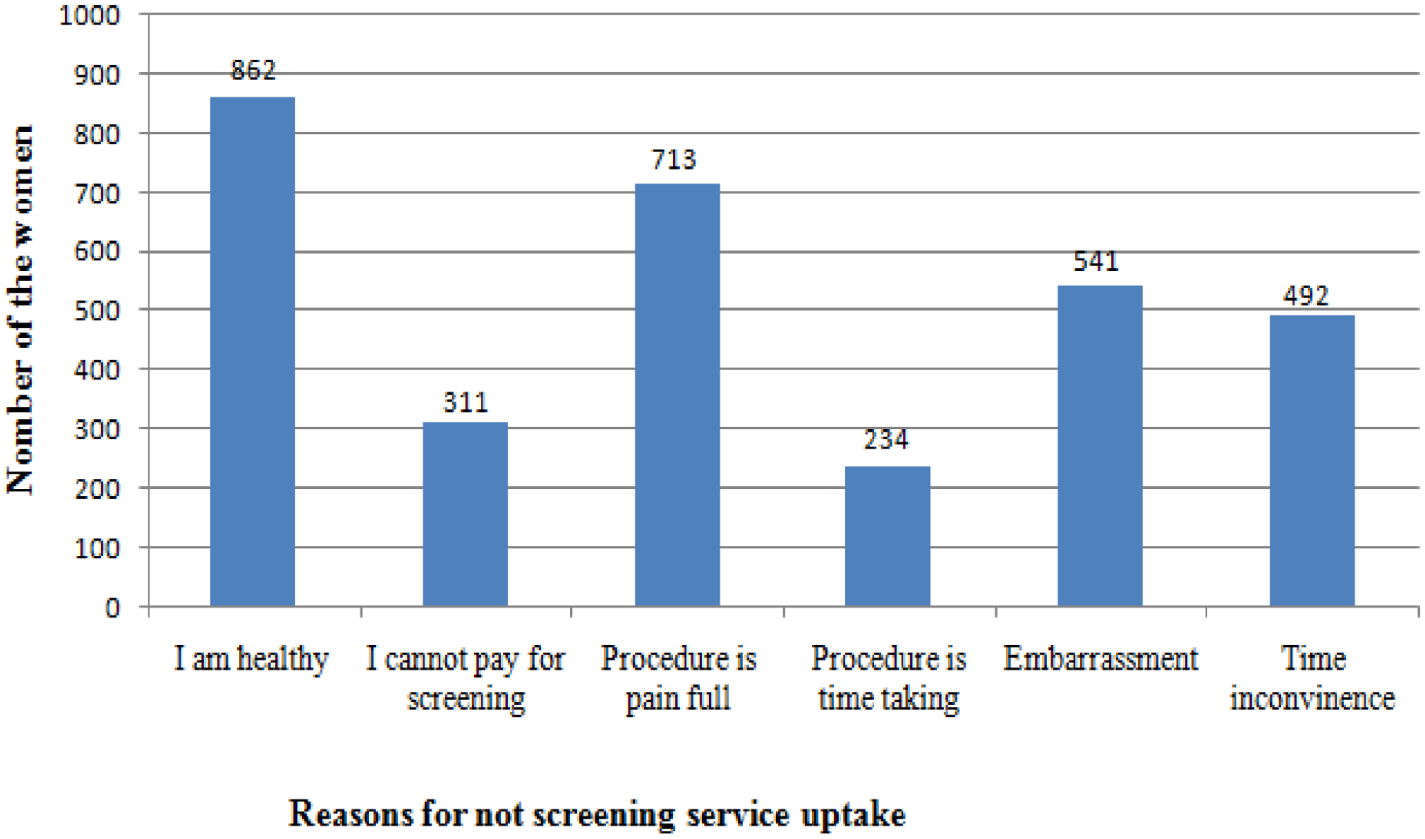
Reasons not to uptake cervical cancer screening service among age eligible women in Mekelle zone, northern Ethiopia, 2015.

The 951 (80.1%) participants who were not screened for cervical cancer were asked for specific reasons that might preclude them from taking the service. The most frequently reported reason for not taking screening service was, feeling of healthiness because of absence symptoms (90.6%), followed by emotional barriers like fear of test procedure is painful (74.9%) (Fig 1).

### Factors Associated with Cervical Cancer Screening Uptake

Bivariate analysis was done to assess any association between individual independent variables and women’s cervical cancer screening service uptake. After controlling the effect of other variables, Age, history of Multiple sexual partners, history of sexually transmitted disease, HIV Sero status, over all knowledge of cancer and screening, Perceived susceptibility to develop cancer and perceived barriers continued to be significantly associated with cervical cancer screening service uptake **(P-values<0.05).**

Women in the age range of 30–39 years were about 1.789 times more likely to be screened for cervical cancer compared with those 21–29 years old (AOR = 1.799, 95%CI = 1.182–2.739) and women who have history of multiple sexual partners were 1.635 times more likely to undergo screening when compared to those who have no history of multiple sexual partner (AOR = 1.635, 95%CI = 1.094–2.443).

Women who have ever experienced sexually transmitted disease were about 1.635 times more likely to undergo cervical cancer screening when compared to those who have not experienced the disease (AOR = 1.635,95%CI = 1.094–2.443).

Women who have been tested positive for HIV were 5.614 times more likely to be screened when compared with those who had tested negative (AOR = 5.614, 95%CI = 2.595–12.144).

Women who have receptive perception of their potential susceptibility to develop cervical cancer were 2.225 times more likely to be screened when compared to those who have non-receptive perception (AOR = 2.225, 95%CI = 1.308–3.783). On the other hand women who have no perceived barriers were 2.256 times more likely to be screened than those who have perceived barriers (AOR = 2.256, 95%CI = 1.447–3.517).

Women who were generally knowledgeable on cervical cancer screening were 2.355 times more likely to undergo screening when compared to those who were not knowledgeable (AOR = 2.355, 95%CI = 1.155–4.802) (Table 4).

**Table 4:** Bivariate and Multivariate analysis of factors associated with cervical cancer screening service uptake among age eligible women in Mekelle zone, northern Ethiopia, june, 2015.

Factors	Screening		Crude ORs(95%CI)	Adjusted ORs(95%CI)
	No	Yes		
**Age of pregnant women**				
21–29	548	66	1	1
30–39	309	106	2.848 (2.032–3.992)	1.799(1.182–2.739)
40–49	52	29	4.631(2.750–7.797)	2.013(.995–4.070)
> = 50	42	34	6.722(3.999–11.298)	2.008(.882–4.573)
**Marital status**				
Single	161	12	1	
Widowed	37	30	10.878(5.093–23.234)	
Divorce	131	47	4.814(2.452–9.450)	
Married	622	146	3.149(1.705–5.817)	
**Occupation**				
Housewife	449	101	1	
Self-employees	286	93	1.446(1.051–1.987)	
Government employees	142	39	1.221(.806–1.849)	
Others	74	2	.120(.029-.498)	
**House hold income**				
<900	252	53	1	
900–1600	256	46	.854(.555–1.315)	
1600–2700	227	64	1.341(.893–2.011)	
>2700	216	72	1.585(1.064–2.361)	
**Age at first sex**				
< = 16	145	62		
>17	806	173	.502(.357-.705)	
**Multiple sexual**				
No	686	119		
Yes	265	116	2.523(1.884–3.380)	1.635(1.094–2.443)
**STD**				
No	912	158		
Yes	39	77	11.396(7.483–17.355)	4.129(2.281–7.476)
**HIV test**				
No	61	7		
Yes	890	228	2.232(1.008–4.946)	
**Sero status**				
Negative	875	177		
Positive	14	51	18.008(9.755–33.244)	5.614(2.595–12.144)
**Instrumental delivery**				
No	524	137		
Yes	230	87	1.447(1.061–1.973)	
**Knowledge**				
Not knowledgeable	193	14		
Knowledgeable	758	221	4.038(2.733–5.966)	2.355(1.155–4.802)
**Perceived susceptibility**				
None receptive	376	30		
Receptive	575	205	4.468(2.982–6.697)	2.225(1.308–3.783)
**Perceived severity**				
None receptive	39	2		
Receptive	912	233	4.982(1.194–20.782)	
**Perceived Barrier**				
Yes	645	83		
No	306	152	3.860(2.859–5.211)	2.256(1.447–3.517

## Discussion

The study revealed that only 235(19.8%) of age eligible women have been screened for cervical cancer. The current cervical cancer-screening uptake (19.8%) is higher when compared to previous, 2008 reports from Ethiopia, where only less than 1% of age eligible women were screened [12]. This might be due to an intervention that has been taking place since 2008 where awareness creation about the screening is being advocated in the country. Currently, the government is expanding the screening centers which might have increased screening service uptake. Screening itself has become a routine procedure for gynecologic patients and is part of the standard care for women who are diagnosed as HIV positive. Recently, there is also a campaign of to screen all HIV positive women who are on ART who were not screened before.

Again, the current screening service uptake is higher than similar reports from Uganda (7%) and India (12%) [13, 14, 15]. This may partly be due to difference in the socio-demographic characteristics of the subjects of the studies. All or some participants of the former studies were rural women who have limited knowledge and access to screening service.

On the contrary, the current utilization of screening service is lower than screening service utilization even by rural women of Kilimanjaro region of Tanzania. The finding showed that 22.6% of the rural Kilimanjaro women had obtained cervical cancer screening. The difference might be due to the fact that the rural area of Kilimanjaro region is one of the catchment areas of a charity organization, the Christian Medical Center and Reproductive Health Centre for cervical cancer screening services working on cervical cancer screening for women aged 18–69 years [16].

According to the current study, the main reason of not taking cervical cancer screening service is absence of symptoms. The same result was also reported from the study done in China which reported no symptoms/discomfort” were among the top three reasons for refusing cervical cancer screening [17]

From this study it was found that maternal age is one of the significant predictors of cervical cancer screening uptake. Women in their 30’s were 1.8 times more likely to be screened compared to women in their 20s (AOR = 1.799, 95%CI = 1.182–2.739). The lower screening rates among younger (21–29 years) women is not unique to Ethiopia; there are also researches with the same findings from elsewhere in Africa and developed countries. According to the study done in one African country, women in the age range of 35–39 were 3 times more likely to be screened compared to those in their 20s [18]. There are also researches with the same findings from Australia, which showed that all other age groups of women years were less likely to have a screening test compared with women aged 30–39 [19]. The explanation for this could be that cervical cancer has bimodal distribution, one at 30s and other at 60s. These two age groups generally become symptomatic to cervical lesion. Therefore, an individual woman sees herself as being at risk and seeks care after recognizes symptoms and perceive susceptibility. Finally, willingness to undergo gynecological examination and screening is performed more likely during their thirties [20, 21, 22].

Another finding of the present study is that history of multiple sexual partners is also an important predictor of cervical cancer screening uptake. Women who have admitted having a recent history of multiple sexual partners were 1.635 times more likely to undergo screening compared to those who did not have such history (AOR = 1.635, 95%CI = 1.094–2.443). This result is consistent with the findings of a study done on the association between sexual behavior and cervical cancer screening in one study in Africa where women who had recent history of multiple sexual partners were more likely to be screened than those who did not have such history [19]. The same result was also reported from Botswana, where women with history of sexually transmitted diseases were 1.66 times more likely to undergo the screening than those without STDs [23].

Sexually transmitted diseases in general and HIV sero status in particular have been found strongly associated with cervical cancer screening uptake. Women who have been diagnosed for sexually transmitted diseases in general were 4 times more likely to be screened than those who have no history of sexually transmitted disease (AOR = 4.129, 95%CI = 2.281–7.476). Particularly, HIV sero-positive women were 5.6 times more likely to consume screening service than HIV sero-negative women (AOR = 5.614, 95%CI = 2.595–12.144). The result is supported by a study done in Botswana and Zambia, which reported that sero positive women were 1.97 and 2.62 more likely to be screened than sero negative women respectively [23,24].

The above association could be explained by strong relationship between multiple sexual partners, increased risk of sexually transmitted diseases like HIV and symptoms as well. The more sexual partners a woman has, the greater are her chances of becoming infected with human immune deficient virus and other sexually transmitted disease like human Papilloma virus, the most common risk factors for development of the cervical cancer. The more the risk, the more women become symptomatic for sexually transmitted disease. Once the women develop symptoms for any of sexually transmitted disease, there is an increased chance of seeking medical help.

Women’s perception about potential susceptibility to cervical cancer was another critical factor in predicting probability of screening uptake. Participants who have receptive perception about potential susceptibility to develop cervical cancer were about 2 times more likely to undergo screening than those who have non-receptive perception (AOR = 2.225, 95%CI = 1.308–3.783).

This result is similar to the findings of a community-based study done in Uganda. The study showed respondents who said they were at risk of developing cervical cancer were 2 times more likely to seek screening for cervical cancer compared with those who believe they had a low risk [25]. Our current finding is also supported by a recent qualitative study on barriers to cervical cancer screening among ethnic minority women, which reported that perceived low risk was one of the practical barriers for cervical cancer screening [12]. This could be explained by the assumption of behavioral model. "The model assumes that belief and attitudes of people are critical determinants of their health-related actions" [21]. It holds that when cues to actions are present, the variations in uptake behavior can be accounted for by beliefs concerning four sets of variables. These include: The individual’s view of own vulnerability to illness. If an individual does not see him or herself as being at risk of any problem, he or she will not seek care [16, 26].

Another important variable from constructs of health belief model is perceived barrier to cervical cancer screening. Women who have not perceived barriers to cervical cancer screening were 2 times more likely to be screened than those who have perceived barriers (AOR = 2.256, **95%CI** = 1.447–3.517). The result is in line with a study conducted in Nigeria and Jamaica which concluded that perceived barriers have significant impact on cervical cancer screening uptake. For example, according to study done on Factors affecting uptake of cervical cancer screening among clinic attendees in Jamaica, women with perceived barriers to cervical cancer screening were 76% less likely to avail of screening when compared to those who have no perceived barriers [21, 27].

The current result is also confirmed by another study done in southern Ghana which revealed that not having screening had significant associations with all seven barrier questions at the 5% Significance level [28]. The theory-based study done among university students in South Africa showed that students who had a Papanicolaus test showed a significantly lower score in barriers to being screened compared to students who did not have the test [29]. This may be due to the fact that perceived barriers of specific health problems are more powerful in affecting health care-seeking behavior [26].

The current research showed women’s knowledge is also implicated in screening uptake. Women who were knowledgeable on cervical cancer and its screening were about 2.355 times more likely to avail of screening services than women who were not knowledgeable **(AOR = 2.355, 95%CI = 1.155–4.802).** Consistent finding was also reported from study done in Tanzania and Botswana which found that knowledge of cervical cancer and its prevention increases the odds of screening uptake about 9 and 3 times respectively [16, 23]. Furthermore, one of the studies done in China reported that women who were willing to undergo screenings had higher knowledge levels [17].

## Conclusions

The study revealed the magnitude of cervical cancer screening service uptake among age eligible women is still unacceptably low.

Common reasons given by women for not undergoing screening were feeling of healthiness because of absent symptoms followed by emotional barriers like fear of test procedure is painful and embarrassment.

Age of the women, history of multiple sexual partners and sexually transmitted disease, HIV Sero-positive, Knowledge, Perceived susceptibility and Perceived Barrier were important predictors of cervical cancer screening service uptake.
